# First-in-Human Randomized Controlled Pilot Trial of a Maxillofacial Drug Delivery Platform for Low-Dose Systemic Delivery

**DOI:** 10.7759/cureus.103916

**Published:** 2026-02-19

**Authors:** Sajani Ramachandran, Ravichandran Kandasamy, Kavita Verma, Pillai Lekshmi Ashokan, Jayahshri Murugan, Jishnu Sudhakar, Hridwik Adiyeri Janardhanan, Anoop UR

**Affiliations:** 1 Dentistry, Pondicherry Institute of Medical Sciences, Puducherry, IND; 2 Biostatistics, Pondicherry Institute of Medical Sciences, Puducherry, IND; 3 Research and Development, UR Anoop Research Group, Puducherry, IND; 4 Computer Science, School of Engineering and Technology, Pondicherry University, Puducherry, IND; 5 Electronics and Communication Engineering, Sri Manakula Vinayagar Engineering College, Puducherry, IND; 6 Computer Science and Engineering, Sri Manakula Vinayagar Engineering College, Puducherry, IND

**Keywords:** area under the plasma concentration–time curve, dose-normalized area under the concentration–time curve, high-performance liquid chromatography, maxillofacial drug delivery, randomized controlled trial

## Abstract

Background

This randomized controlled trial (RCT) evaluated a novel maxillofacial drug delivery platform for safe, rapid, and controlled systemic delivery of a low dose of metronidazole through a maxillofacial route that bypasses gastrointestinal absorption and first-pass hepatic metabolism.

Methods

In this parallel-group, pilot RCT (n = 20), patients undergoing maxillary first molar endodontic therapy received either 400 mg of oral metronidazole (control group; n = 10) or 5 mg of metronidazole administered using the maxillofacial platform (study group; n = 10). Sixty blood samples were collected at baseline, 15 minutes, and 30 minutes to assess early systemic exposure. Three turbid samples from one patient in the study group were excluded, resulting in nine patients in the study group, 10 in the control group, and 57 plasma samples for high-performance liquid chromatography (HPLC) analysis. Plasma metronidazole concentrations were quantified using HPLC, and the area under the plasma concentration-time curve (AUC) was calculated to assess systemic drug exposure. Due to non-normal data distribution, non-parametric statistical analyses were performed. Local and systemic adverse events (AEs) were monitored throughout the study.

Results

Primary analysis using dose-normalized AUC (DN AUC) demonstrated significant early systemic exposure following maxillofacial administration, despite an 80-fold lower dose compared to oral delivery. Sensitivity analysis using raw AUC values showed comparable systemic exposure between the two groups, supporting the robustness of the findings. No local or systemic AEs were observed in either group.

Conclusion

The maxillofacial drug delivery platform demonstrates potential for safe and rapid systemic drug delivery at low doses.

## Introduction

Efficient, safe, and targeted drug delivery technologies are essential for maximizing therapeutic efficacy while minimizing adverse effects [1,2]. Conventional routes of drug administration, such as enteral, parenteral, transdermal, nasal, pulmonary, ocular, and vaginal, are limited by challenges ranging from enzymatic degradation in the gastrointestinal tract to restricted transport across biological barriers [3]. As the therapeutic use of nucleic acids, proteins, and antibodies is increasing in clinical practice, the need for novel drug delivery strategies that can bypass these barriers is becoming increasingly crucial [4,5].

Therefore, a drug delivery platform that can provide painless, repeated, and controlled drug administration for different types of drugs by bypassing the gut and first-pass hepatic metabolism holds immense potential.

This pilot RCT study aimed to evaluate whether a novel maxillofacial drug delivery platform could safely and rapidly deliver metronidazole into systemic circulation using a very low dose (5 mg) by bypassing the gut and first-pass metabolism. The plasma concentrations of metronidazole following maxillofacial administration were compared with those obtained after oral administration of a standard dose (400 mg) at 15 minutes and 30 minutes post-administration.

A version of this manuscript was published as a preprint in medRxiv as "A Novel Maxillofacial Technology for Drug Administration-A Randomized Controlled Trial Using Metronidazole" (medRxiv 2024.09.11.24313269; doi: https://doi.org/10.1101/2024.09.11.24313269).

## Materials and methods

Trial design

The trial was designed as a parallel-group randomized controlled trial. The Institutional Ethics Committee of Pondicherry Institute of Medical Sciences gave ethical approval (IEC: RC/2022/169). Consenting adult patients with carious maxillary first molars involving the pulp who reported to the dental outpatient department at Pondicherry Institute of Medical Sciences, India, were recruited for the study. The study was registered prospectively with the ICMR Clinical Trial Registry - India as CTRI/2023/03/050279.

Participants and study methods

Adult patients reporting to the dental outpatient department with carious maxillary first molars with infected pulps were examined and recruited for the study. Between 10/03/2023 and 10/03/2024, 75 patients were screened, and 20 consenting patients who fulfilled the inclusion criteria were randomly assigned to either the control group (n = 10) or to the study group (n = 10) using a computer-generated sequence of random numbers.

The control group consisted of patients in whom a single 400 mg dose of metronidazole was given orally after biomechanical preparation of the root canals. The study group consisted of patients in whom 5 mg of metronidazole solution was administered into the pulp cavity of a tooth using the maxillofacial technique after biomechanical preparation of the root canals.

**Figure 1:**
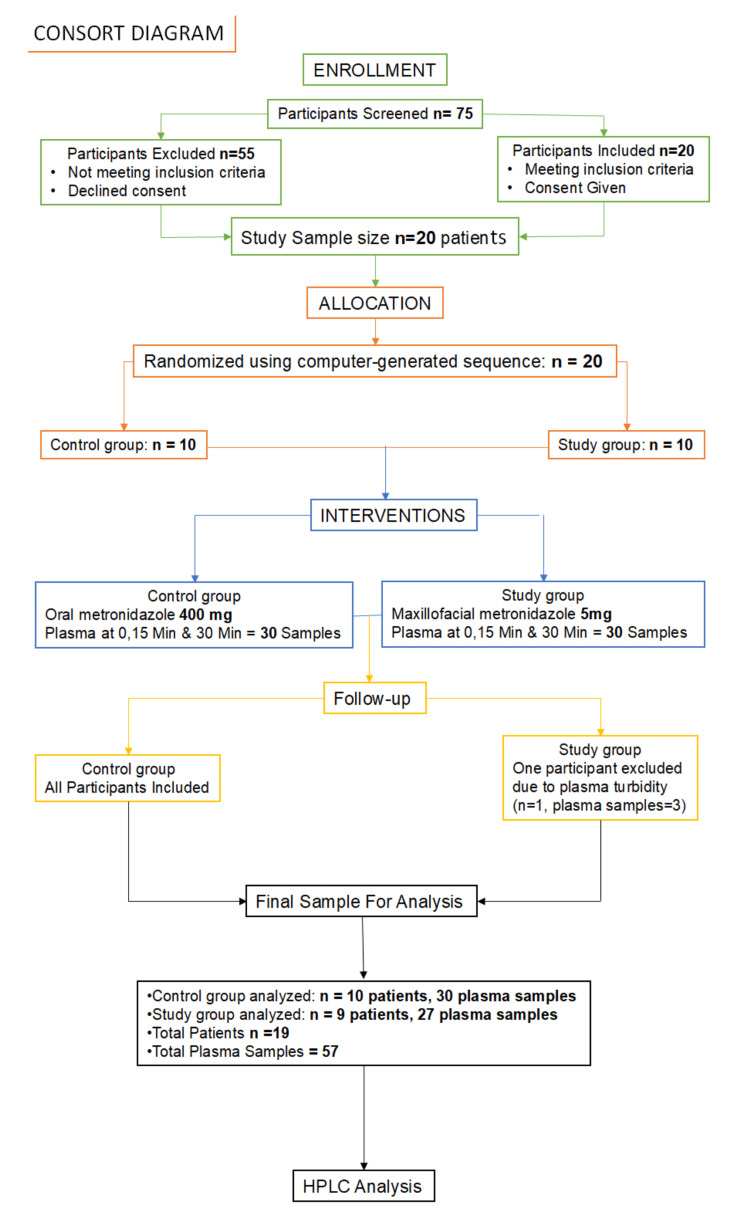
Consort diagram

An almost equal number of male and female patients (11 males and nine females) were recruited for the study. The mean age of the patients in the control group was 40.5 ± 14.3 years, and in the experimental group, it was 44.1 ± 11.6 years. Although 10 patients were initially allotted to each group, the plasma samples of one patient in the study group could not be analyzed due to turbidity and were therefore excluded from high-performance liquid chromatography (HPLC) analysis. As a result, the study group ultimately consisted of nine patients whose samples were analyzed. The consort diagram is shown in Figure 1.

Inclusion criteria

Patients aged 18 to 60 years presenting with clinical and radiographic findings indicative of pulpal involvement in a maxillary first molar and requiring root canal treatment were included in the study. Only systemically healthy individuals who were not on any regular medications and had no contraindications to root canal therapy were considered eligible for participation.

Exclusion criteria

Patients presenting with periapical lesions in maxillary first molars not associated with pulpal involvement were excluded from the study. Patients who had taken metronidazole or any other drugs within the previous 72 hours were also excluded. Individuals with a known allergy to metronidazole were excluded. In addition, patients with bleeding disorders, hepatic or renal disease, or those receiving antiplatelet therapy were excluded from the study.

Randomization and masking

Patients were randomly assigned to either the control group or the study group using a computer-generated random number sequence. Allocation concealment was ensured through sequentially numbered, sealed, opaque envelopes. Patients and the operating dentist were not masked to the drug administration procedure. But the outcome assessor was blinded.

Procedure/interventions

Carious upper first molars with pulpal involvement were selected, and root canal treatment was initiated under local anesthesia. After opening the access to the pulp chamber, the pulp canals were located, and the pulp was extirpated. Biomechanical preparation of the root canals was performed with standard rotary files, and irrigation was done with saline solution.

In the control group, the access opening to the pulp cavity was closed with zinc oxide eugenol paste, and each patient received a single oral dose of 400 mg of metronidazole in tablet form.

**Figure 2:**
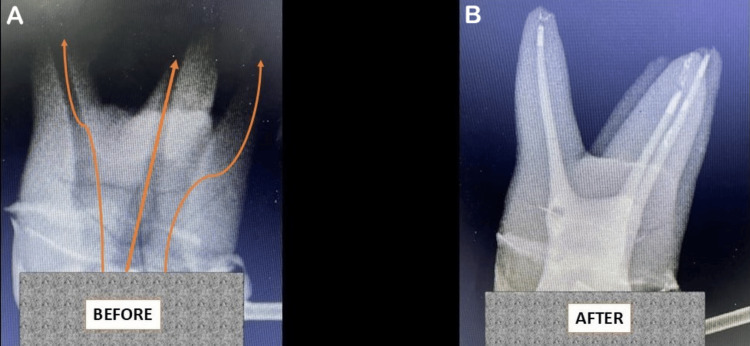
Representative radiographic images of a maxillary first molar before and after in vitro administration of a radiopaque solution using the maxillofacial drug delivery technology (A) Pre-administration radiograph demonstrating baseline anatomy of the pulp cavity of the maxillary first molar, arrows indicate the drug exit route from the pulp cavity. (B) Post-administration radiograph showing radiopaque root canals, demonstrating the path of flow.

In the study group, after extirpation of pulp and widening of the pulp canals, the canals were irrigated with saline and dried. Tooth preparation was done, and a preliminary impression was made for a temporary crown. The crown was then fabricated for use with the maxillofacial drug delivery technology. The maxillofacial drug delivery system was temporarily secured to the tooth, and metronidazole infusion solution containing 500 µg/100 µl was delivered into the pulp cavity in a controlled manner for 10 minutes at the rate of 100 µl/min. After drug administration, the maxillofacial drug delivery system was removed, and the access opening to the pulp cavity was temporarily closed with zinc oxide eugenol cement. All dental procedures and drug administration were done by the same operator to avoid inter-operator bias. The drug delivery route in an in vitro tooth specimen is shown in Figure 2. 

All 20 patients were followed up for one month to monitor adverse events such as pain, swelling, or infection at the treated tooth site. Patients were advised to report to the dental outpatient department weekly during the follow-up period. They were also advised to report any adverse event by mail or phone whenever any such events occurred. None of the 20 patients reported pain, swelling, or infection at the treated tooth site.

Blood sample collection

Blood samples (n = 60) from both groups (control group: 10, study group: 10) were collected before starting the root canal treatment. Following drug administration, blood samples (3 ml) were collected at 15 minutes (control group: 10, study group: 10) and 30 minutes (control group: 10, study group: 10) by an experienced technician using sterile EDTA vacutainer tubes. In one patient in the study group, the samples (n = 3) were found to be turbid and were excluded from the study. As a result, the study group consisted of only nine patients. A total of 57 plasma samples were analyzed for the presence of metronidazole. The plasma was isolated by centrifuging at 3000 rpm for 5 minutes. The isolated plasma was stored at -80°C until dispatch. The collected plasma was dispatched in dry ice- compatible shipping boxes to the HPLC laboratory for analysis.

Outcomes

The primary outcomes were early detection of metronidazole in systemic circulation at 15 minutes and 30 minutes after maxillofacial administration of 1/80th of the oral dose, comparison of the proportion of patients with detectable plasma metronidazole between the maxillofacial and oral groups, and comparison of plasma exposure between the two routes at the same time points, by measuring the area under the plasma concentration-time curve (AUC) using HPLC analysis.

Statistical analysis

Sample Size Determination

To detect an effect size of 1.3 in plasma metronidazole levels between the control group (oral administration) and the study group (maxillofacial drug delivery), with a 5% significance level and 80% power, the required sample size was estimated at nine patients per group. Accounting for a 10% attrition rate, the sample size was increased to 10 per group. The effect size of 1.3 was estimated from Stolz et al. [6].

Statistical Methods

Continuous variables were summarized using medians with interquartile ranges (IQRs), while categorical variables were described as frequencies and percentages. Due to the small sample size, non-normal distributions, and the presence of values below the limit of detection, non-parametric statistical methods were applied. Binary outcomes were compared using Fisher’s exact test for between-group analyses and McNemar’s test for within-group comparisons over time, where applicable. Continuous outcomes were compared between the oral and maxillofacial routes using the Mann-Whitney U test.

Early systemic exposure was assessed using AUC at 15 and 30 minutes. Because of the marked difference in the administered dose between the two routes, dose-normalized (DN)-AUC was used for the primary analysis to allow fair comparison of the administration routes. In the primary analysis, values below the limit of detection (LOD) were imputed as LOD/2 to avoid bias from non-detectable samples and to retain all subjects in the analysis. A sensitivity analysis was performed using raw AUC values to evaluate the robustness of the primary findings. Samples below the LOD were excluded from the sensitivity analysis. Effect sizes were reported using Cliff’s δ with 95% confidence intervals.

IBM SPSS Statistics for Windows, Version 29.0 (released 2022, IBM Corp., Armonk, NY), was used for the statistical data analysis. The statistical alpha significance level was considered to be at the 0.050 level.

## Results

A total of 57 plasma samples (pre-administration: 0 minutes; post-administration: at 15 minutes and 30 minutes) from both groups were analyzed for the presence of metronidazole.

All pre-administration plasma samples in both groups were negative for metronidazole.

In the control group, following oral administration of 400 mg, metronidazole was detected in five (50%) patients. In the study group, following maxillofacial administration of 5 mg (1/80th of the oral dose), the drug was detected in all nine (100%) patients. This difference was statistically significant (p = 0.033). The absolute difference in the proportion of patients with detectable metronidazole between the two groups was 0.50 (95% CI: 0.101-0.763) (Table 1).

**Table 1:** Comparison of metronidazole detection in plasma following oral versus maxillofacial administration

Status	Oral tablet group, n (%)	Maxillofacial route group, n (%)	p-value
Present	5 (50)	9 (100)	0.033
Absent	5 (50)	0 (0)	

When comparing the post-administration plasma samples at 15 minutes and 30 minutes after oral administration, metronidazole was detected in four (40%) plasma samples at 15 minutes and in five (50%) plasma samples at 30 minutes. This difference was not statistically significant (p = 1.0) (Table 2).

**Table 2:** Comparison of plasma metronidazole detection at different time points within the oral administration group

Time/status	30 minutes		Total	p-value
	Present	Absent		
15 minutes				
Present	4	0	4	1.000
Absent	1	5	6	

Following maxillofacial administration, metronidazole was detected in five (55.6%) plasma samples at 15 minutes and in 7seven (77.8%) plasma samples at 30 minutes. Of the five positive plasma samples at 15 minutes, only three remained positive at 30 minutes. This difference was not statistically significant (p = 0.687) (Table 3).

**Table 3:** Comparison of plasma metronidazole detection at different time points within the maxillofacial administration group

Time/status	30 minutes		Total	p-value
	Present	Absent		
15 minutes				
Present	3	2	5	0.687
Absent	4	0	4	

Plasma metronidazole concentrations were measured using HPLC with an LOD of 1000 AU (absorbance units). Due to the 1:80 dose difference between the maxillofacial (5 mg) and oral (400 mg) routes, the primary analysis was performed by calculating DN-AUC for all patients. DN-AUC was calculated as the area under the concentration-time curve (AUC) in AU*min (absorbance units x minutes) divided by the administered dose. All values below the LOD were imputed as LOD/2 (500 AU) to allow inclusion of all patients. Pooled ranks were calculated separately at each time point using DN-AUC values pooled across both administration routes, and tied values were assigned the average rank (Table 4).

**Table 4:** Individual pharmacokinetics and dose-normalized area under the concentration-time curve (DN AUC) at 15 and 30 minutes (primary analysis; LOD/2 inclusive) LOD: limit of detection, DN-AUC: dose-normalized area under the concentration-time curve

Route	Subject	Dose (mg)	AUC 15 min	DN-AUC 15 min	LOD/2?	Included in sensitivity?	AUC 30 min	DN-AUC 30 min	LOD/2?	Included in sensitivity?
Oral	1	400	500	1.25	Yes	No	500	1.25	Yes	No
Oral	2	400	500	1.25	Yes	No	2,402	6.01	No	Yes
Oral	3	400	92,305	230.76	No	Yes	301,085	752.71	No	Yes
Oral	4	400	7,598	18.99	No	Yes	43,320	108.3	No	Yes
Oral	5	400	6,065	15.16	No	Yes	5,306	13.27	No	Yes
Oral	6	400	18,641	46.6	No	Yes	40,157	100.39	No	Yes
Oral	7	400	500	1.25	Yes	No	500	1.25	Yes	No
Oral	8	400	500	1.25	Yes	No	500	1.25	Yes	No
Oral	9	400	500	1.25	Yes	No	500	1.25	Yes	No
Oral	10	400	500	1.25	Yes	No	500	1.25	Yes	No
Max	1	5	11,371	2,274.20	No	Yes	18,673	3,734.60	No	Yes
Max	2	5	500	100	Yes	No	80,396	16,079.20	No	Yes
Max	3	5	500	100	Yes	No	38,863	7,772.60	No	Yes
Max	4	5	32,418	6,483.60	No	Yes	14,938	2,987.60	No	Yes
Max	5	5	500	100	Yes	No	1,748,290	349,658.00	No	Yes
Max	6	5	1,087,239	217,447.80	No	Yes	500	100	Yes	No
Max	7	5	42,239	8,447.80	No	Yes	47,373	9,474.60	No	Yes
Max	8	5	13,852	2,770.40	No	Yes	500	100	Yes	No
Max	9	5	500	100	Yes	No	161,621	32,324.20	No	Yes

For the primary analysis, medians and interquartile ranges (IQRs) were calculated, including LOD/2-imputed values. Between-route comparisons at each time point were conducted using the Mann-Whitney U test with exact p-values. Effect size was quantified using Cliff’s δ with corresponding 95% confidence intervals (Table 5).

**Table 5:** Early dose-normalized area under the concentration-time curve (DN-AUC) by route of administration (primary analysis; LOD/2 inclusive) LOD: limit of detection, DN-AUC: dose-normalized area under the concentration-time curve

Time point	Route	n (subjects)	Plasma samples < LOD	Median DN-AUC	IQR	Cliff’s δ (95% CI)	Mann–Whitney U	Exact p-value
15 min	Oral	10	6 / 10	1.25	1.25–18.04	—	—	—
	Max	9	4 / 9	2,274.2	100.0–6,483.6	0.91 (0.58–1.00)	4.0	0.00074
30 min	Oral	10	5 / 10	3.63	1.25–78.61	—	—	—
	Max	9	2 / 9	7,772.6	2,987.6–16,079.2	0.87 (0.52–1.00)	6.0	0.00151

**Figure 3:**
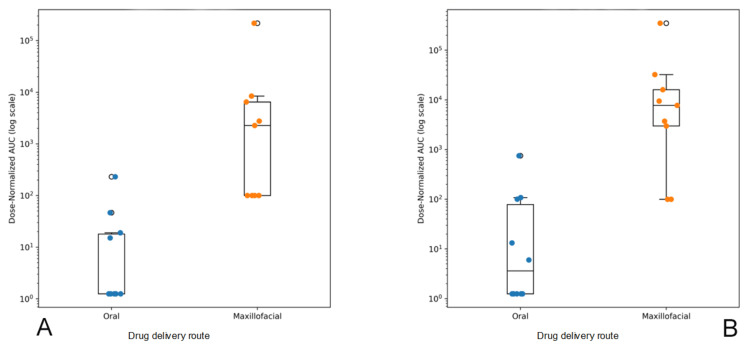
Dose-normalized area under the concentration-time curve (DN-AUC) at 15 and 30 minutes following oral and maxillofacial administration (log scale) Boxplots show the median and interquartile range, with whiskers representing the full data range. Individual data points, jittered horizontally for clarity, represent subject-level DN-AUC values. The y-axis is presented on a logarithmic scale to accommodate the wide range of exposure values. and better visualization. (A) 15 minutes. (B) 30 minutes.

At 15 minutes, the median DN-AUC was 1.25 (IQR 1.25-18.04) following oral dosing and 2,274.2 (IQR 100.0-6,483.6) following maxillofacial delivery. At 30 minutes, the median DN-AUC increased to 3.63 (IQR 1.25-78.61) in the oral group and 7,772.6 (IQR 2,987.6-16,079.2) in the maxillofacial group. Rank-based comparisons between routes at each time point demonstrated significantly higher early exposure with maxillofacial delivery (15 minutes: Mann-Whitney U = 4.0, p = 0.00074; 30 min: U = 6.0, p = 0.00151), with large effect sizes (Cliff’s δ = 0.91 at 15 minutes and 0.87 at 30 minutes), indicating a high probability that DN-AUC values were greater in the maxillofacial group. Boxplots depicting median, interquartile range, and individual DN-AUC values at 15 and 30 minutes following oral and maxillofacial administration are shown in Figure 3.

For the sensitivity analysis, raw AUC values without normalization were used. All values below the LOD were excluded. Only subjects with measurable plasma concentrations were included in the rank calculations. Ranks were pooled across routes and assigned separately at each time point using only the included values. This sensitivity analysis was conducted to evaluate the robustness of the primary findings with respect to the handling of below-LOD values (Table 6).

**Table 6:** Individual early raw AUC at 15 and 30 minutes (sensitivity analysis, LOD excluded) AUC, LOD: limit of detection, AUC: area under the concentration-time curve

Route	Subject	Dose (mg)	Raw AUC 15 min	LOD/2?	Included in sensitivity?	Raw AUC 30 min	LOD/2?	Included in sensitivity?
Oral	1	400	500	Yes	No	500	Yes	No
Oral	2	400	500	Yes	No	2,402	No	Yes
Oral	3	400	92,305	No	Yes	301,085	No	Yes
Oral	4	400	7,598	No	Yes	43,320	No	Yes
Oral	5	400	6,065	No	Yes	5,306	No	Yes
Oral	6	400	18,641	No	Yes	40,157	No	Yes
Oral	7	400	500	Yes	No	500	Yes	No
Oral	8	400	500	Yes	No	500	Yes	No
Oral	9	400	500	Yes	No	500	Yes	No
Oral	10	400	500	Yes	No	500	Yes	No
Max	1	5	11,371	No	Yes	18,673	No	Yes
Max	2	5	500	Yes	No	80,396	No	Yes
Max	3	5	500	Yes	No	38,863	No	Yes
Max	4	5	32,418	No	Yes	14,938	No	Yes
Max	5	5	500	Yes	No	1,748,290	No	Yes
Max	6	5	1,087,239	No	Yes	500	Yes	No
Max	7	5	42,239	No	Yes	47,373	No	Yes
Max	8	5	13,852	No	Yes	500	Yes	No
Max	9	5	500	Yes	No	161,621	No	Yes

For the sensitivity analysis, medians and interquartile ranges (IQRs) were calculated using included values only. Between-route comparisons at each time point were performed using the Mann-Whitney U test with exact p-values. Cliff’s δ was reported as a non-parametric measure to quantify effect size. 95% confidence intervals were calculated and may include zero, indicating no statistically significant difference between routes in the sensitivity analysis (Table 7).

**Table 7:** Early raw exposure by route of administration (sensitivity analysis, LOD excluded) LOD: limit of detection, AUC: area under the plasma concentration-time curve

Time point	Route	n (subjects)	Plasma samples < LOD	Median AUC	IQR	Cliff’s δ (95% CI)	Mann–Whitney U	p-value
15 min	Oral	4	6 / 10	13,119.5	7,214.8–37,057.0	—	—	—
	Max	5	4 / 9	32,418.0	13,852.0–42,239.0	0.40 (−0.34 to 0.86)	6.0	0.413
30 min	Oral	5	5 / 10	40,157.0	5,306.0–43,320.0	—	—	—
	Max	7	2 / 9	47,373.0	28,768.0–121,008.5	0.31 (−0.36 to 0.78)	12.0	0.432

At 15 minutes, four oral-route patients and five maxillofacial-route patients had measurable AUCs. The median AUC was 13,119.5 (IQR: 7,214.8-37,057.0) for oral administration and 32,418.0 (IQR: 13,852.0-42,239.0) for the maxillofacial route, with a moderate effect size (Cliff’s δ = 0.40; 95% CI: −0.34 to 0.86) and no statistically significant difference (Mann-Whitney U = 6.0; p = 0.413). At 30 minutes, five oral-route patients and seven maxillofacial-route patients had measurable AUCs. Median AUC was 40,157.0 (IQR: 5,306.0-43,320.0) for oral and 47,373.0 (IQR: 28,768.0-121,008.5) for the maxillofacial route, with a small to moderate effect size (Cliff’s δ = 0.31; 95% CI: −0.36 to 0.78) and no statistically significant difference (Mann-Whitney U = 12.0; p = 0.432).

**Figure 4:**
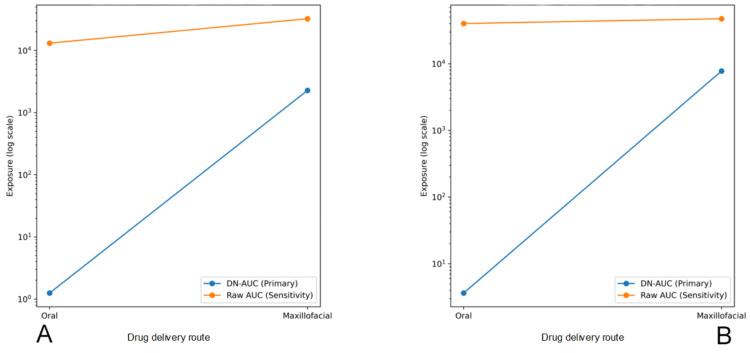
Direction of early systemic exposure between primary and sensitivity analyses at 15 and 30 minutes (A) 15 minutes: both analyses demonstrate higher exposure following maxillofacial administration, indicating consistent directionality despite differences in scaling and dose normalization. (B) 30 minutes: dose-normalized exposure remains higher following maxillofacial administration, while raw AUC values show a smaller difference between oral and maxillofacial routes, reflecting convergence of absolute exposure as absorption progresses at the higher oral dose. AUC: area under the plasma concentration-time curve

**Figure 5:**
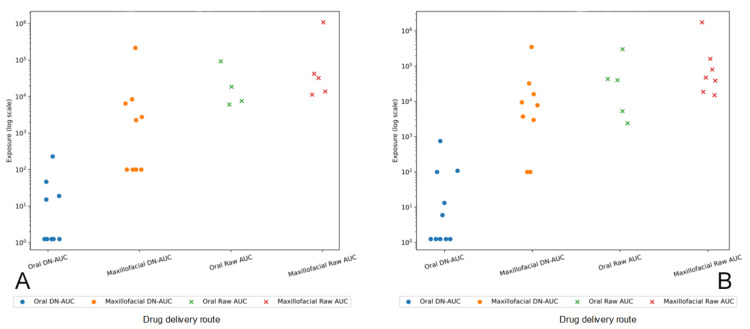
Individual exposure directionality at 15 and 30 minutes Individual subject exposure values are shown for oral and maxillofacial delivery using both DN-AUC and raw AUC analyses. Each point represents one subject, with horizontal jitter for visualization. Data are displayed on a logarithmic scale. (A) 15 minutes: individual DN-AUC and raw AUC values are consistently higher following maxillofacial delivery compared with oral administration, indicating similar directionality at the individual level across primary and sensitivity analyses. (B) 30 minutes: DN-AUC values remain higher following maxillofacial delivery, whereas the difference in raw AUC values between the routes is smaller. DN AUC: dose-normalized area under the concentration-time curve

Median and individual plasma exposure at 15 and 30 minutes following oral and maxillofacial administration are shown for both the primary DN-AUC analysis and the raw AUC sensitivity analysis in Figure 4 and Figure 5, respectively.

Relative systemic exposure between the routes further highlights the magnitude of these differences. Relative exposure was calculated descriptively as the ratio of median DN-AUC values between the maxillofacial and oral routes. The IQR-based relative exposure range was estimated using the ratio of the maxillofacial first quartile to the oral third quartile (lower bound) and the ratio of the maxillofacial third quartile to the oral first quartile (upper bound). These values are descriptive and were not subjected to formal statistical testing (Table 8).

**Table 8:** Descriptive relative systemic exposure by route of administration

Timepoint	Oral DN AUC, median (IQR)	Maxillofacial DN AUC, median (IQR)	Median Relative Exposure (MF / Oral)	IQR-based relative exposure range
15 min	1.25 (1.25–18.04)	2,274.2 (100.0–6,483.6)	1,819-fold	5.5–5,187
30 min	3.63 (1.25–78.61)	7,772.6 (2,987.6–16,079.2)	2,141-fold	38.0–12,863

At 15 minutes, the maxillofacial route produced a median relative exposure of 1,819-fold compared with oral administration, with an IQR-based range of 5.5-5,187-fold. At 30 minutes, the median relative exposure increased to 2,141-fold (IQR-based range: 38.0-12,863-fold). These findings indicate substantially higher early systemic exposure via the maxillofacial route, although variability across subjects was considerable.

Overall, both primary and sensitivity analyses demonstrate that early plasma exposure is consistently higher following maxillofacial administration than following oral administration, with the relative exposure analysis showing the large magnitude of this difference. 

## Discussion

Novel drug delivery technologies have become game changers in clinical practice with the advent of newer drug formulations in the form of nucleic acids, peptides, proteins, and antibodies. These technologies, along with newer routes for drug administration, are now crucial for delivering drugs across challenging biological and physiological barriers.

Conventional routes of drug delivery, like the enteral, parenteral, transdermal, nasal, pulmonary, ocular, and vaginal routes, have limitations [3]. Therefore, there is a need for drug delivery technologies and routes that can provide painless, repeated, and controlled drug administration for different types of drugs at low doses.

In clinical practice, medicaments are locally placed in the pulp cavities of teeth to eliminate bacteria before obturation of root canals. Some studies have observed that intracanal medicaments such as formocresol can enter systemic circulation through blood vessels present in the residual radicular pulp following pulpotomy.

Pulpotomy is a dental procedure that involves surgical amputation of only the coronal part of vital pulp and placement of a dressing over the exposed healthy pulp stumps. Formocresol is widely used for pulpotomy. Studies have shown that in children receiving pulpotomy under general anesthesia, there was an increase in blood levels of formocresol from the baseline in a small number of samples after application of formocresol to the root stumps for five minutes [7]. Similarly, studies using radiolabeled paraformaldehyde that was applied to root stumps in rhesus monkeys and dogs have shown distribution to regional lymph nodes, blood, kidneys, and liver [8].

By contrast, pulpectomy or root canal procedure involves the complete removal of pulp from both the crown and root part of a tooth. The pulp cavity of such a root canal-treated tooth is completely devoid of any blood vessels. Existing studies based on root canal-treated teeth are, therefore, limited to comparisons of the efficacy of intracanal versus systemic antibiotics for infection control and the role of endodontic pathogens in systemic disease.

To date, no randomized controlled clinical trial has used a completely pulp extirpated, root canal-treated tooth as a portal for intentional, painless, repeated, and controlled systemic drug delivery. In this world's first pilot clinical trial, a maxillofacial drug delivery platform was temporarily secured to a pulp extirpated upper molar, and a drug was administered into the pulp cavity of the tooth in a controlled manner.

This pilot study evaluated whether the maxillofacial drug delivery platform could safely deliver metronidazole into the systemic circulation in a controlled manner, following administration of a low 5 mg dose of metronidazole solution (1/80th of the conventional 400 mg oral dose) through the pulp cavity of a root canal-treated maxillary first molar. The presence of metronidazole in plasma following maxillofacial administration was further compared with that observed after oral administration of a standard 400 mg dose.

In this study, two significantly different doses of the same drug (5 mg versus 400 mg) were administered through two different routes (maxillofacial platform-assisted drug administration versus Oral administration). Metronidazole was chosen for administration because the study population consisted of patients requiring root canal treatment. Infection of the root canal system is polymicrobial. Intracanal medicaments are used in infected root canals to eliminate residual bacteria after root canal instrumentation. In addition, metronidazole has predictable linear pharmacokinetics, high bioavailability, low inter-subject variability, well-characterized absorption, distribution, and metabolism, an established safety profile, and is available in different formulations.

Metronidazole, a nitroimidazole antibiotic with a broad spectrum, is effective against obligate anaerobic bacteria. It is bactericidal for anaerobic organisms even at very low concentrations of 0.5%. The advantage of using metronidazole in dentistry as an intracanal agent includes ready availability, rapid bactericidal action, good tissue penetration, cost effectiveness, acceptable pharmacokinetics and pharmacodynamics, sustained antimicrobial activity, and the inability of susceptible organisms to develop resistance. Additionally, metronidazole can be safely re-administered if necessary [9].

In a study evaluating the antibacterial activity of 0.12% chlorhexidine gel, 10% metronidazole gel, calcium hydroxide plus distilled water, calcium hydroxide along with camphorated para-monochlorophenol, and calcium hydroxide along with glycerine using an agar diffusion test, it was found that metronidazole caused inhibition of the growth of all the tested obligate anaerobes [10]. Furthermore, when metronidazole was used as an intracanal medicament in infected primary teeth, the success rate was 83% [11]. Metronidazole, when used as an intracanal medicament in infected primary molars, had a success rate of 85% after 24 months [12]. A study evaluating the efficacy of metronidazole gel versus metronidazole solution against Enterococcus faecalis in abscessed primary molars found that metronidazole gel (3% w/v) was more effective than metronidazole solution (0.5 % w/v) against *E. faecalis* [13]. Studies have shown that the minimum inhibitory concentration of metronidazole for most anaerobes, including *Bacteroides fragilis*, is less than 6 µg/ml [14,15]. In a study, after an oral dose of 250 mg of metronidazole, the peak serum level of the drug was found to be 6.2 µg/ml, and after an oral dose of 500 mg of metronidazole, the peak serum level was 11.2 µg/ml. Similarly, after a single oral dose of 2 g of metronidazole, the peak serum level was 40 µg/ml [15].

Metronidazole has also been extensively studied using different delivery routes such as oral, intravenous, rectal, vaginal, and topical application in the oral cavity. Oral and intravenous routes provide the most efficient systemic delivery, whereas rectal, vaginal, and topical application in the oral cavity yield low plasma concentrations [16,17].

Oral administration demonstrates high bioavailability, with rapid absorption, peak plasma concentrations within one to two hours, and an elimination half-life ranging from six to 14 hours. Intravenous administration provides complete systemic exposure, with pharmacokinetic parameters comparable to oral dosing. Rectal suppositories exhibit lower bioavailability of 53-54% and a time of approximately 4.9 hours to achieve peak concentration, indicating less efficient systemic absorption. Vaginal formulations yield markedly reduced systemic exposure [16,17]. In a study, intravaginal gel produced only about 2% of the systemic exposure associated with a 500 mg oral dose, exhibited a bioavailability of only 56%, and took a prolonged time of approximately eight hours to achieve peak concentration [18]. Topical application in the oral cavity, such as gels or rinses for periodontal or gingival infections, achieves high localized concentrations with minimal systemic absorption, making them suitable for targeted local therapy but ineffective for systemic delivery [6]. These findings highlight the limitations of existing delivery routes and underscore the potential value of maxillofacial administration, which achieved detectable systemic concentrations at only 1/80th of the conventional oral dose in this study.

Metronidazole belongs to the Biopharmaceutical Classification System Class 1. It is a highly soluble and highly permeable drug. It has >90% bioavailability. Metronidazole exhibits linear and time-independent pharmacokinetics across oral, intravenous, rectal, and intravaginal routes. Hence, plasma concentration increases proportionally with dose, and absorption, metabolism, distribution, and elimination remain consistent over time. However, the absolute systemic exposure (AUC and C max) of metronidazole can vary between routes due to differences in bioavailability and absorption, even though the proportionality and time-independence are maintained. Peak plasma concentrations are detected at one to two hours following oral administration. The plasma protein binding and volume of distribution are 10-15% and 0.51-1.1 L/kg, respectively. The drug is metabolized in the liver, and the kidney is the major route of excretion [16,17]. In this study, these pharmacokinetic characteristics of metronidazole support the plausibility of achieving detectable plasma concentration following maxillofacial administration despite the markedly low administered dose.

In this pilot study, metronidazole was detectable in the plasma of all patients (9; 100%) following the maxillofacial platform- assisted administration of 5mg, whereas only half of the patients 5 (50%) exhibited detectable levels of metronidazole after oral administration of 400 mg. Fisher’s exact test revealed a statistically significant difference between the two routes of administration (p = 0.033), indicating a non-random association between route and systemic drug detectability. The markedly higher and earlier detection of metronidazole after maxillofacial administration, despite the substantially lower dose, may be evidence that this route provides a more rapid systemic uptake than conventional oral delivery. Further, the observed effect may be due to the bypassing of the gastrointestinal tract and hepatic first-pass metabolism, in addition to the functionality of the maxillofacial drug delivery platform. These findings highlight the potential of the maxillofacial platform-assisted drug administration as a reliable alternative for achieving systemic drug exposure at very low doses.

Following oral administration, metronidazole was detected in 4 samples at 15 minutes and 5 samples at 30 minutes. Following maxillofacial administration, the drug was detected in 5 samples at 15 minutes and 4 samples at 30 minutes, with only 3 samples positive at both time points. In both routes, some patients exhibited detectable plasma levels at one time point but not the other. Within each route, McNemar’s test was used to assess changes in the detectability of metronidazole between 15 and 30 minutes. No statistically significant within-route changes in detectability were observed.

The observed variability can be attributed to the expected inter-individual variability in early drug distribution. Differences in gastrointestinal absorption in the case of oral administration, regional blood flow in the case of maxillofacial administration, and early distribution kinetics, combined with plasma concentrations near the LOD, can produce transient detectability. These fluctuations can be considered physiological and hence do not indicate inconsistencies in either delivery platform. Importantly, measurable systemic levels were achieved by the maxillofacial platform at a fraction of the oral dose, supporting its potential as a low-dose alternative for systemic delivery.

This exploratory pilot study included plasma metronidazole values that were below the LOD. Therefore, a predefined primary analysis and a complementary sensitivity analysis were used to assess the robustness of early systemic exposure achieved using the maxillofacial drug delivery platform.

The primary analysis included all enrolled patients. DN-AUC values were calculated for each subject to account for the significant difference in administered doses between the oral and maxillofacial routes. These values formed the basis for all primary comparisons of early systemic exposure. LOD/2 was imputed for values below the LOD. This approach is commonly used in early pharmacokinetic investigations to minimize bias and to preserve sample size.

Individual-level primary analysis data from Table 4 demonstrate that, following dose normalization, maxillofacial administration yielded higher AUC values at both 15 and 30 minutes compared with oral administration. This finding was observed despite the markedly lower administered maxillofacial dose. A greater proportion of subjects in the maxillofacial group showed quantifiable plasma concentrations at both 15 and 30 minutes. In contrast, oral administration was associated with a higher frequency of values that were at or below the LOD.

Group-level summaries and rank-based comparisons from Table 5 support these individual-level findings. Median dose-normalized AUC values were significantly higher following maxillofacial platform-assisted delivery at both 15 and 30 minutes. The between-route differences were statistically significant and associated with large effect sizes. The magnitude of Cliff’s δ indicated minimal overlap between exposure distributions, supporting a clear separation between routes that may not be attributable to random variation alone.

From a feasibility perspective, the primary analysis data show that the maxillofacial platform can facilitate early systemic drug delivery at a very low dose. Quantifiable plasma concentrations were detected in the majority of subjects at early time points, supporting effective drug delivery through the maxillofacial route. In this pilot study, preliminary evidence of safety was demonstrated by the absence of any observed or reported acute device or procedure-related adverse events.

Sensitivity analysis based on raw AUC data and excluding values below the LOD Table 6 was done to evaluate whether the primary findings were influenced by LOD/2 imputation. When the analysis was restricted to subjects with quantifiable plasma concentrations, maxillofacial administration continued to demonstrate higher early exposure values than oral dosing at both 15 and 30 minutes. Pooled rank ordering showed that, among included subjects, maxillofacial platform-assisted AUC values were consistently ranked higher, indicating a directional separation between routes which was consistent with the primary analysis findings.

At the same time, the sensitivity analysis presented in Table 6 and Table 7 also highlighted significant inter-individual variability in early systemic exposure, particularly within the maxillofacial group, where raw AUC values spanned several orders of magnitude. The reduced number of analyzable subjects after exclusion of LOD/2 observations resulted in attenuation of statistical significance and wider confidence intervals, consistent with a reduction in power rather than a reversal in the direction of effect. When the sensitivity analysis was restricted to subjects with quantifiable early plasma concentrations and values below the limit of detection were excluded, the direction of the early exposure advantage associated with the maxillofacial platform remained unchanged. Pairwise comparisons of raw AUC values continued to favor maxillofacial platform-assisted delivery at both 15 and 30 minutes. However, the difference between the routes was no longer statistically significant due to reduced sample size and increased inter-individual variability. Cliff’s δ estimates were of moderate magnitude with confidence intervals spanning zero. Further, the higher oral AUC values observed at 30 minutes likely reflect continued absorption consistent with the known linear kinetics of metronidazole following conventional oral administration, and therefore do not contradict the significantly greater dose-normalized early exposure achieved with maxillofacial delivery.

Despite these conservative analytical conditions, higher pooled exposure ranks for maxillofacial administration persisted, indicating that the primary findings were not driven by LOD/2 imputation but rather reflected a clinically meaningful difference in early exposure profiles between routes. Together, these sensitivity analysis findings support the robustness of the primary analysis, demonstrate the feasibility of maxillofacial platform-assisted drug delivery, and highlight the variability inherent in early systemic exposure measurements in a pilot study.

Complementary descriptive analysis of relative early systemic exposure based on dose-normalized AUC values in Table 8 provides additional context for interpreting the magnitude of the between-route differences observed in the primary analysis. Despite administration of only 1/80th of the conventional oral dose, maxillofacial delivery was associated with higher median dose-normalized exposure at both early time points, corresponding to approximately 1,800-fold greater exposure at 15 minutes and more than 2,100-fold greater exposure at 30 minutes relative to oral administration. Although the wide interquartile-based relative exposure ranges reflect expected inter-individual variability in early human pharmacokinetics, the consistently elevated relative exposure underscores the capacity of the maxillofacial platform to achieve rapid systemic availability at very low doses. These findings further support the feasibility of the maxillofacial platform and provide a rationale for further clinical development.

This first-in-human pilot study was designed to assess the feasibility and preliminary safety of the maxillofacial platform-assisted drug delivery for achieving early systemic exposure at an ultra-low dose. Primary and secondary analyses showed that maxillofacial administration of even 1/80th of the standard oral dose resulted in detectable early plasma concentrations. Further, the observed rank dominance and robustness under conservative sensitivity analysis collectively indicate that the maxillofacial platform is capable of facilitating rapid systemic drug delivery in a clinical setting. The absence of observed or reported adverse events supports the preliminary safety of the maxillofacial platform and justifies further evaluation in larger clinical studies.

Post-administration sampling was limited to early-phase exposure, as blood sampling was done only at 15 and 30 minutes. However, the linear pharmacokinetics of metronidazole made these early AUC measurements informative for assessing early systemic uptake. The inter-individual variability in plasma detection likely reflects physiological differences rather than limitations of the delivery platform. Ethical considerations inherent to the first-in-human design and dose minimization constraints limited the extent of sampling. Hence, comprehensive pharmacokinetic studies are required in the future to rigorously compare maxillofacial administration with oral dosing.

This pilot study was not designed to evaluate the complete pharmacokinetic equivalence, bioavailability, or therapeutic efficacy of metronidazole that was administered using the maxillofacial platform. The limited sample size for evaluating the maxillofacial platform, limited early-phase blood sampling, and the pilot design preclude definitive conclusions regarding later-phase exposure, total systemic exposure over time, or inter-route equivalence across the complete pharmacokinetic profile of metronidazole. In addition, the study does not assess pharmacodynamic endpoints, clinical effectiveness of the detected metronidazole concentration, or long-term safety. This study also does not determine optimal dosing strategies or generalizability to other drugs or patient populations. Accordingly, the findings should be interpreted as proof-of-feasibility, providing a rationale for larger, appropriately powered clinical studies with extended sampling and dose-escalation designs.

## Conclusions

This first-in-human pilot study supports the feasibility of maxillofacial platform-assisted systemic drug delivery and warrants further clinical investigation of this technique.

The maxillofacial platform is a cost-effective technology and may be particularly useful for patients with swallowing difficulties, in emergencies requiring rapid systemic delivery, and for patients who require repeated, painless drug delivery on a daily basis. Furthermore, as the maxillofacial technology can be used safely in routine outpatient clinical settings, it may be readily accessible to patients. This clinical trial also highlights the potential of the maxillofacial platform for delivering drugs in neurodegenerative conditions, such as Parkinson’s and Alzheimer’s, as these patients are prone to dental caries due to inadequate maintenance of oral hygiene and may routinely require root canal treatment. Additionally, it may be useful for delivering drugs that are degraded or poorly absorbed in the gastrointestinal tract, such as peptides, proteins, antibodies, and nucleic acid-based therapeutics.
